# Emerging roles and therapeutic implications of lipid droplet protein perilipin 2 in liver disease

**DOI:** 10.1016/j.gendis.2025.101712

**Published:** 2025-06-07

**Authors:** Xuwen Xiang, Jing Chen, Lin Che

**Affiliations:** a Department of Child Health, Department of Pediatrics, Women and Children's Hospital, School of Medicine & School of Public Health, Xiamen University, Xiamen, Fujian 361102, China; b State Key Laboratory of Oncology in South China, Guangdong Provincial Clinical Research Center for Cancer, Sun Yat-sen University Cancer Center, Guangzhou, Guangdong 510060, China

**Keywords:** Lipid droplets, Liver disease, Perilipin 2, Small molecular inhibitors, Therapeutic target

## Abstract

Lipid droplets (LDs) are dynamic organelles that store neutral lipids when energy is in excess and serve as an energy reservoir during energy deprivation. Altered hepatic lipid metabolism is a critical factor influencing the development of liver disease, such as viral hepatitis, fatty liver disease, and hepatocellular carcinoma. Perilipin 2 (PLIN2) is a protein associated with the metabolism of intracellular LDs and is closely related to the clinical outcome of liver disease. While the impact of PLIN2 on the pathogenesis of liver disease is gradually being recognized, the mechanism of action remains unclear. In this review, we highlight recent advances in the understanding of PLIN2's role in the pathogenesis of liver disease through LD biogenesis, LD contact sites, LD dynamics, and lipophagy. Furthermore, we discuss the current opportunities for PLIN2-targeted therapy for liver disease.

## Introduction

Liver disease has emerged as a significant global health concern and economic burden, with approximately two million people dying from liver disease annually.^1^ The use of vaccinations and effective treatments has led to a decline in disability-adjusted life years attributed to liver viruses over the past decade. However, China continues to bear the highest burden of hepatitis B and C infections worldwide.^2^ Furthermore, due to high alcohol consumption, aging, and increased exposure to metabolism-related risk factors, it is anticipated that the mortality associated with alcohol-related liver disease (ALD) and metabolic-associated fatty liver disease (MAFLD) will witness a substantial surge in the forthcoming decades.^2^ Notably, China has the highest incidence rate and associated mortality of MAFLD in Asia and is also experiencing the most rapid growth rate of MAFLD cases among all countries in the world, as estimated between 2016 and 2030, due to urbanization trends.^3^^,^^4^ If this acceleration persists, China will inevitably have the largest number of patients with MAFLD globally, thereby imposing an immense burden on both public health systems and economies worldwide.^3^ Consequently, there is an urgent need to increase our understanding of MAFLD and other liver diseases within China, along with the implementation of control strategies.

The liver serves as the primary site of fat production and lipid oxidation in the human body and functions as the central organ of lipid metabolism.^5^ Disruption of lipid metabolism in the liver causes excess lipids to accumulate, which is a crucial factor contributing to the development of liver disease. Fatty acids in the liver originate from various sources, including direct intake from external sources, *de novo* adipogenesis, and adipose tissue lipolysis.^6^ These accumulated fatty acids can be processed by hepatocyte β- or ω-oxidation into acetyl-CoA or esterified into triglycerides, forming very-low-density lipoprotein, which is subsequently released from the liver.^6^ Dysregulation of liver lipid metabolism leads to excessive fatty acid accumulation within the liver, ultimately resulting in various liver diseases.

Lipid droplets (LDs) are key components of liver lipid metabolism.^7^ LDs are dynamic organelles that store neutral lipids and are widely present in most cells, from yeast to humans, and they play a key role in maintaining cellular lipid and energy homeostasis.^8^ The accumulation of LDs in the liver is a hallmark of MAFLD.^7^ Increased adipogenesis and decreased lipolysis can lead to dysregulated LD metabolism, which in turn promotes hepatic steatosis.^9^ Perilipin 2 (PLIN2) is a protein associated with intracellular LD metabolism; increased expression of PLIN2 is linked to liver disease, including viral hepatitis, fatty liver disease, and hepatocellular carcinoma (HCC). Despite preliminary studies on the role and mechanisms of PLIN2 in these liver diseases, its regulatory network and potential applications remain unclear.

In this review, we aimed to provide a comprehensive overview of the roles and implications of PLIN2 in liver disease. In particular, we highlighted the intimate association between PLIN2 and LDs, and the mechanism by which this facilitates hepatic lipid metabolism. Finally, drawing on recent advances in our understanding of PLIN2 function, we examined the pharmacological approaches targeting this mechanism and their therapeutic potential for treating liver disease.

## Lipid droplet biogenesis and its contact sites

**Figure 1:**
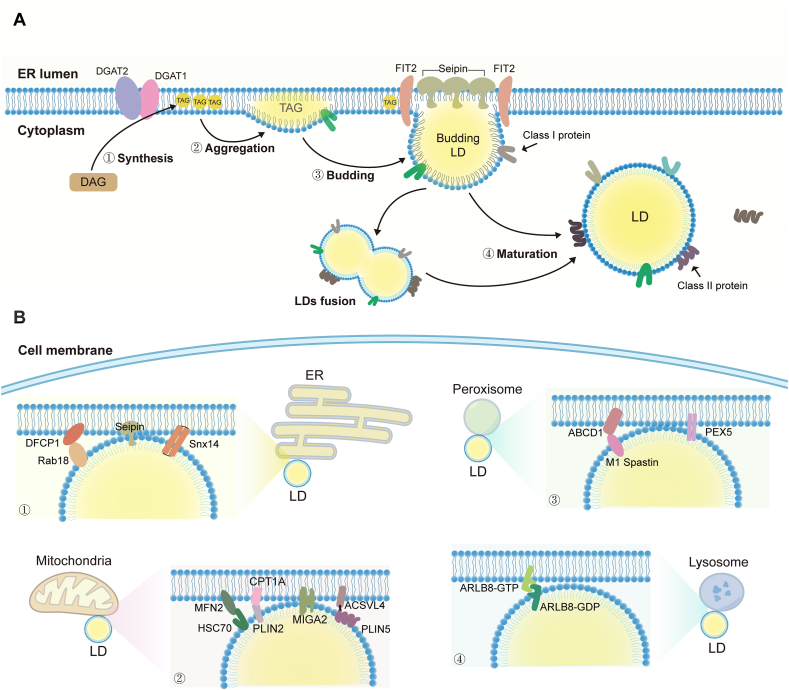
LD biogenesis and its contact sites. **(A)** The formation of cytoplasmic LDs in mammals involves four steps: neutral lipid synthesis, neutral lipid aggregation, new LD budding, and LD maturation. LD-associated proteins are generally classified into class I proteins from the endoplasmic reticulum (ER) and class II proteins (*e.g.*, perilipins). **(B)** Cytoplasmic LDs can interact with intracellular organelles such as the ER, mitochondria, peroxisome, and lysosome. ABCD1, ATP binding cassette subfamily D member 1; ACSVL4, acyl-CoA synthetase long chain family member 4; ARL8B, ADP-ribosylation factor-like protein 8B; CPT1A, carnitine palmitoyltransferase 1A; DAG, diacylglycerol; DFCP1, double FYVE containing protein 1; DGAT, diacylglycerol transferase; FIT2, fat storage-induced transmembrane 2; HSC70, heat shock cognate protein; LDs, lipid droplets; MIGA2, mitoguardin 2; Snx, sorting nexin; TAG, triacylglycerol.

LDs are formed in the endoplasmic reticulum in a process by which neutral lipids form a hydrophobic core that is surrounded by a single layer of phospholipid molecules, with various LD-related proteins distributed on the surface (Fig. 1A).^10^ Triacylglycerol serves as an exemplar for inducing LD formation. In the endoplasmic reticulum, diacylglycerol transferase 1 (DGAT1) and DGAT2 catalyze the conversion of diacylglycerol into triacylglycerol.^8^ Once neutral lipids accumulate beyond 2.8–10.0 mol% triacylglycerol, they coalesce to form a lens.^9^ Seipin and other factors involved in LD biogenesis are recruited to this lens to facilitate the growth of nascent LDs,^11^, ^12^ a process that is also facilitated by fat storage-induced transmembrane 2 (FIT2).^8^ Following budding, LDs expand through triacylglycerol synthesis or fusion with other LDs, ultimately maturing and detaching from the endoplasmic reticulum membrane.^8^

After that, LDs are distributed in the cytoplasm and nucleus, where they protect cells by storing lipids, thus reducing the negative effects that excess fatty acids and sterols exert on the cell membrane composition, signaling, and metabolic homeostasis.^13^ Cytoplasmic LD can also establish contact sites with various organelles (*e.g.*, endoplasmic reticulum, mitochondria, peroxisomes, and lysosomes) to participate in lipid exchange and transport between organelles and regulate organelle dynamics (Fig. 1B).^14^ The interaction between LD and the endoplasmic reticulum facilitates the formation of new LDs, with this linkage primarily mediated by seipin.^15^ Sorting nexin 14 (Snx14) serves as a marker for endoplasmic reticulum-LD contacts.^16^ Additionally, the interaction between the endoplasmic reticulum-localized double FYVE-containing protein 1 (DFCP1) and the LD-localized Rab18-ZW10 complex promotes the formation of endoplasmic reticulum-LD contacts.^17^ Fatty acids released by LD lipolysis enter mitochondria through an interaction site with LD, where they undergo β-oxidation to produce energy. The mitochondrial outer membrane protein mitoguardin 2 (MIGA2) links mitochondria to LD.^18^ Acyl-CoA synthetase long-chain family member 4 (ACSVL4) interacts with perilipin 5 (PLIN5), mitochondrially localized mitofusin-2 (MFN2), and LD-localized heat shock cognate protein 70 (HSC70), while perilipin 2 (PLIN2) and the mitochondrial outer membrane protein carnitine palmitoyltransferase 1A (CPT1A) form complexes that are key to tethering mitochondria to LD.^19^, ^20^, ^21^ These interactions facilitate mitochondrial contact with LD and are essential for their tethering. Peroxisomes are capable of oxidizing long-chain fatty acids, while LDs store lipids that can be utilized by peroxisomes. The M1 spastin protein forms a tethering complex with the peroxisomal protein ATP-binding cassette subfamily D member 1 (ABCD1), thereby promoting contact between LD and peroxisome.^22^ Furthermore, peroxisomes and peroxisomal biogenesis factor 5 (PEX5) facilitate fasting-induced lipolysis by stimulating the translocation of adipose triglyceride lipase (ATGL) to LD.^23^ The lysosome plays a critical role in the degradation of LD, particularly during autophagy. The GDP-bound form of ADP-ribosylation factor-like protein 8B (ARL8B) binds to LD, while the GTP-bound form localizes to lysosomes.^24^ The GDP- and GTP-bound states form complexes that facilitate contact between LDs and lysosomes, mediating lipid transfer.^24^

## Functions of lipid droplet protein PLIN2

**Figure 2:**
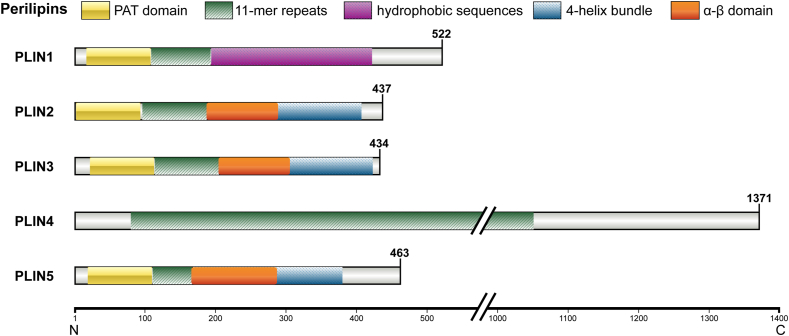
Structure of human perilipins. Perilipins usually consist of an N-terminal PAT domain, a central region containing 11-mer repeats, and a variable C-terminal domain. PLIN4 only has an elongated N-terminal region with 29 tandem 33-mer repeats; PLIN1 has hydrophobic sequences at its C-terminus; PLIN2/3/5 contain a 4-helix bundle and an additional α-β domain. PAT, process analytical technology.

In mammalian cells, the LD proteome consists of 100–150 proteins, whereas in yeast, it comprises 35–40 proteins. The LD proteome is dominated by enzymes involved in lipid metabolism, including members of the perilipin family. The perilipin family, also known as the PAT family, consists of five proteins, numbered 1–5, in the order in which they were discovered.^25^ These include perilipin (PLIN1), adipocyte differentiation-associated protein (ADRP) or adipophilin (PLIN2), mannose-6-phosphate binding protein (PLIN3), plasma membrane-associated protein (PLIN4), and myocardial lipid droplet protein (PLIN5).^26^ PLINs are typically composed of three distinct protein domains: an N-terminal PAT domain, a central region containing 11-mer repeats that form amphiphilic helices, and a variable C-terminal domain, while PLIN2 possess a unique α-β domain in C-terminus (Fig. 2).^27^^,^^28^ PLIN2, discovered in 1992, is involved in the production, maturation, and degradation of LDs.^29^ The human *PLIN2* gene is located at 9p22.1–p22.3, with a total gene length of 5003 bp, and encodes the 50 kDa protein PLIN2, comprising 437 amino acids, with a highly conserved PAT domain located at amino acids 1–115 in the N-terminal region.^30^ The PAT domain is associated with the maintenance of LD stability, lipid accumulation, and proteasomal degradation of PLIN2. The N-terminal 11-mer repeats domain located at residues 103–215 was shown to interact with the C-terminal domain to form adaptive connections between LDs and the plasma membrane to regulate milk fat secretion.^31^ The PLIN2 C-terminal region contains an α-β domain that forms a conserved lipid-binding cleft with helix α6, while its 4-helical bundle domain exhibits high lipid affinity.^31^

PLIN2 is widely present in human fat, liver, lung, breast, muscle, and other tissues and is generally expressed in all cells,^32^ particularly in liver cells. Activation of peroxisome proliferator-activated receptor gamma (PPARγ) induces PLIN2 expression,^33^^,^^34^ and a high-fat diet stimulates an increase in PLIN2 expression via PPARγ, thereby promoting triacylglycerol storage in adipocyte LDs.^35^ In the *PLIN2*-encoded variant Ser251Pro (rs35568725), the serine at residue 251 at the C-terminus is mutated to proline, which disrupts the α-helix, resulting in reduced levels of triglycerides and very-low-density lipoprotein in human plasma.^36^ PLIN2 protein is degraded by the ubiquitin-proteasome pathway,^37^ and ubiquitin protein ligase E3 component n-recognin 1 (Ubr1) targets PLIN2 for degradation in an amino acid-dependent manner.^38^ PLIN2 bound to LD membranes can also be degraded by companion-mediated autophagy (CMA), in which PLIN2 binds to HSC70 and then interacts with lysosome-associated membrane protein type 2A (LAMP-2A), leading to the degradation of PLIN2.^39^ Phosphorylation of PLIN2 by adenosine monophosphate-activated protein kinase (AMPK) enhances CMA degradation.^40^

**Figure 3:**
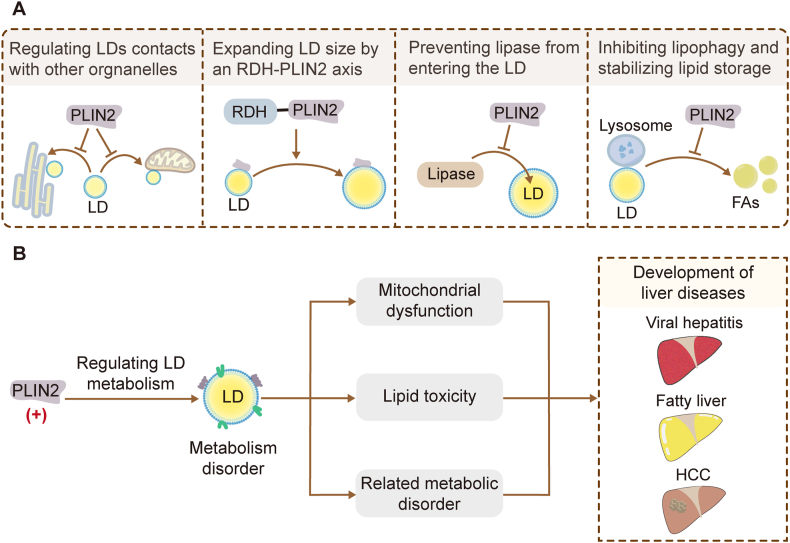
Functions of PLIN2 in LDs. **(A)** PLIN2 can regulate the contacts between LDs and other organelles. PLIN2 can be recruited to small lipid droplets by the endoplasmic reticulum protein RDH to expand LD size. PLIN2 can prevent lipase from entering the LD and destroying the LD stability. PLIN2 can also inhibit LD lipophagy and stabilize lipid storage. **(B)** Overexpression of *PLIN2* can lead to LD metabolism disorders, which in turn lead to impaired mitochondrial function, lipid toxicity, and related metabolic disorders, eventually leading to the development of liver disease such as viral hepatitis, fatty liver, and HCC. PLIN2, perilipin 2; FA, fatty acid; HCC, hepatocellular carcinoma; LD, lipid droplet; RDH, retinol dehydrogenase.

PLIN1 and PLIN2 are distributed on the surface of LDs and can be used as signature structural proteins to identify LDs.^41^ The association of PLIN2-deficient LDs with marker proteins of several membrane structures, including the endoplasmic reticulum, mitochondria, and lysosomes, is significantly increased compared with that of the normal PLIN2 variant, producing enhanced interactions between LDs and intracellular organelles (Fig. 3A).^42^ Mutual stabilizations between PLIN2 and LDs regulate LD dynamics and their relationship with mitochondria.^42^ When expressed under lipogenic conditions, human hepatocytes carrying the PLIN2 Pro251 variant become heavily loaded with small, uniform LDs.^43^ LD size is regulated by the retinol dehydrogenase (RDH)-PLIN2 axis.^44^ LD catabolism that frees fatty acids involves two pathways: lipolysis and lipophagy. Hepatocytes use cytoplasmic lipase and lipophagy pathways to degrade large and small LDs, respectively.^45^ PLIN2 prevents lipases from entering LDs and inhibits lipophagy to stabilize lipid storage in LDs.^25^^,^^45^^,^^46^

During dynamic interactions between LDs and mitochondria, fatty acids produced by LD lipolysis translocate into the mitochondria for β-oxidation under certain nutritional conditions, and LD metabolism disorders can impair mitochondrial function and cause lipid toxicity and related metabolic disorders, leading to various liver-related diseases, including viral hepatitis, fatty liver, and HCC.^47^ PLIN2 mediates the relationship between LDs and mitochondria by regulating LD metabolism,^42^ and disorders of this ultimately lead to the occurrence and development of liver disease (Fig. 3B).

## PLIN2 in liver disease: roles and mechanisms

**Figure 4:**
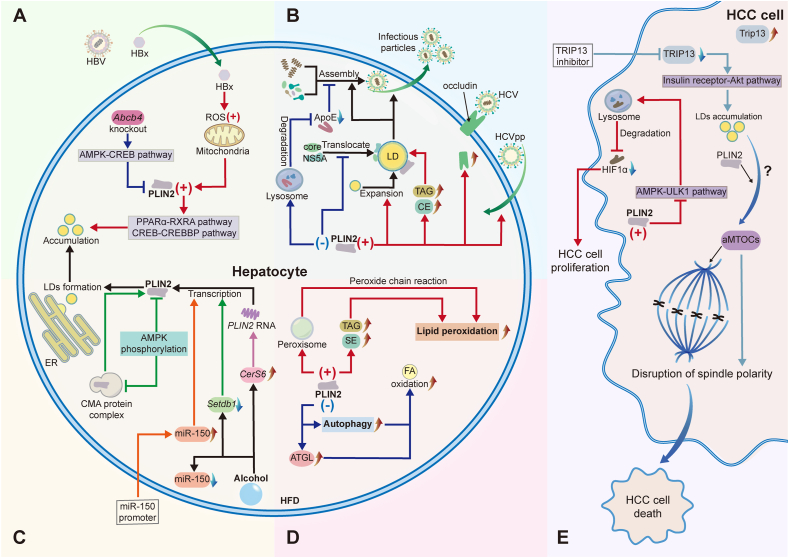
Role of PLIN2 in liver disease. **(A)** After HBx enters hepatocytes, mitochondrial ROS increases, which boosts PLIN2 expression, leading to the formation and accumulation of LDs through PPARα-RXRA and CREB-CREBBP pathways. Knockout of *Abcb4* could inhibit PLIN2 expression and alleviate LD accumulation through the AMPK-CREB pathway. **(B)** In HCV-infected hepatocytes, higher PLIN2 levels increase the surface area of LD, providing a platform for more efficient HCV assembly. PLIN2 also promotes the total content of TAG and CE, aiding in the production of infectious viral particles, and increases HCV pseudo particle (HCVpp) entry and occludin expression. Inhibiting PLIN2 affects the transport of core and NS5A to the LD surface, hindering virus assembly initiation, and accelerates ApoE degradation by lysosomes, impacting virus assembly. **(C)** Alcohol can lead to CerS6 up-regulation, Setdb1 down-regulation, and compensatory down-regulation of miR-150 in cells. CerS6 up-regulation may enhance PLIN2 RNA stability, increase PLIN2 levels, promote LD formation, and promote alcoholic steatosis. Setdb1 down-regulation increases PLIN2 active transcription, inhibits AMPK phosphorylation, disrupts the formation of the CMA protein complex that captures PLIN2, stabilizes PLIN2 protein, and prevents LD degradation. miR-150 up-regulation could activate PLIN2 transcription and promote LD accumulation. **(D)** The increase in intracellular PLIN2 under HFD causes TAG and SE accumulation or promotes peroxisomal sequestration of unwanted or harmful lipids through oxidative chain reactions, leading to increased lipid peroxidation. Knockdown of PLIN2 could promote ATGL and autophagy to significantly increase FA oxidation. **(E)** PLIN2 increases in HCC cells, which inhibits the activity of the AMPK/ULK1 pathway, thereby delaying the degradation of HIF1α by lysosomes and promoting the proliferation of HCC cells. Trip13 expression increases in HCC cells, and inhibition of Trip13 can promote LD accumulation through the insulin receptor/Akt pathway. In the presence of PLIN2, through some unknown mechanism, LDs may convert into aMTOCs, destroy spindle polarity in mitosis, and lead to tumor cell death. Akt, protein kinase B; aMTOCs, acentric microtubule organizing centers; AMPK, adenosine monophosphate-activated protein kinase; Abcb4, ATP binding cassette subfamily B member 4; ApoE, apolipoprotein E; ATGL, adipose triacylglyceride lipase; CE, cholesteryl ester; CerS6, ceramide synthase 6; CMA, companion mediated autophagy; ER, endoplasmic reticulum; FA, fatty acid; HBx, hepatitis B virus X protein; HCC, hepatocellular carcinoma; HCV, hepatitis C virus; HFD, high-fat diet; HIF1α, hypoxia-inducible factor alpha; LDs, lipid droplets; PLIN2, perilipin 2; PPARγ, peroxisome proliferator-activated receptor γ; ROS, reactive oxygen species; SE, sterol ester; Setdb1, SET domain bifurcated 1; TAG, triacylglycerol; Trip13, thyroid hormone receptor factor 13; Ubr1, ubiquitin protein ligase E3 component n-recognin 1; ULK1, Unc-51-like autophagy-activating kinase 1; PPARα, peroxisome proliferator-activated receptor alpha; RXRA, retinoid X receptor alpha; CREB, cAMP-response element binding protein; CREBBP, CREB-binding protein.

Recent studies have fully discussed the important role of LDs in liver disease and have proposed the possibility of treating liver disease by regulating LDs. However, as an important LD protein that regulates LD metabolism, PLIN2 has not been comprehensively explored in the pathogenesis of various liver diseases or in its significance as a therapeutic target (Fig. 4).

## Hepatitis B

Hepatitis B virus (HBV) is an enveloped DNA virus with a DNA genome comprising relaxed circular DNA (rcDNA) of approximately 3.2 kb in length, with one complete minus strand and one incomplete plus strand. The viral genome encodes four overlapping open reading frames C, P, S, and X, encoding the functional proteins HBc, HBe, Pol, HBs, and HBx, respectively.^48^ Wang et al demonstrated that HBx transfection in Huh7/MIHA cells increased mitochondrial reactive oxygen species, promoting LD formation, with N-acetylcysteine reversing this effect.^49^ This study specifically examined HBx effects in isolation, without establishing a complete HBV infection model. Elevated intracellular reactive oxygen species levels up-regulate PLIN2 expression in hepatocytes through oxidative stress, and the increased PLIN2 then disrupts lipid homeostasis by both suppressing peroxisome proliferator-activated receptor alpha (PPARα)-retinoid X receptor alpha (RXRA) signaling to reduce fatty acid oxidation and enhancing cAMP-response element binding protein (CREB)-CREB-binding protein (CREBBP) activity to promote lipogenesis, ultimately driving hepatic lipid accumulation (Fig. 4A).^50^ Therefore, increased mitochondrial reactive oxygen species in HBV-infected cells may promote LD formation by up-regulating PLIN2 expression, leading to liver lipid accumulation and corresponding pathological changes. Cholestasis resulting from knockout of the ATP-binding cassette subfamily B member 4 (*Abcb4*) transporter gene modulated the expression of PLIN2 through the activation of the AMPK-CREB signaling pathway, thereby reducing lipid accumulation in the livers of HB mice (Fig. 4A).^51^ This further suggests that we can regulate the level of PLIN2 by controlling the *Abcb4* expression to improve liver lesions caused by HBV infection.

## Hepatitis C

Hepatitis C virus (HCV) is a single-loop positive-stranded RNA virus with a gene sequence that encodes various structural and nonstructural proteins. The structural proteins include the nucleocapsid protein (or core protein) and two envelope glycoproteins (E1 and E2 proteins), and the nonstructural proteins include p7, NS2, NS3, NS4A, NS4B, NS5A, and NS5B.^52^ The relationship between the HCV replication cycle *in vivo* and LD metabolism has been extensively studied. The life cycle of HCV in host cells is closely related to LD metabolism, and its replication, assembly, and release in host cells require the cooperation of intracellular LDs.^53^ LD metabolism is regulated by the surface of the inlaid LD proteins, which interacts with HCV viral proteins to promote the production of HCV infectious progeny.

Branche et al found that HCV Jc1 RNA transfection in Huh-7 cells increased both PLIN2 protein and mRNA levels compared with untransfected controls.^54^ While PLIN2 overexpression reduced intracellular HCV core RNA levels and particle infectivity, it increased the amount of secreted virus and its infectivity through three late-stage mechanisms: expanding LD surface area for virion assembly, increasing triglycerides and cholesteryl ester content for infectious viral particle formation, and up-regulating occludin (an essential HCV receptor) expression to promote the entry of HCV pseudo particles.^54^ This stage-dependent duality reflects PLIN2's distinct roles in early replication inhibition versus late assembly/egress facilitation. However, in a Huh7 cell model infected with derived HCV (HCVcc, JFH-1 strain), the expression of *PLIN2* was down-regulated, whereas forced expression of miR-148a and miR-30a at the mRNA and protein levels induced *PLIN2* expression, potentially because the two miRNAs inhibited HCV, minimizing PLIN2 displacement from the LD surface and protecting LDs from proteomic degradation.^55^ Transfection of the same cells with JFH1 and Jc1 produced divergent changes in PLIN2 levels, which was potentially attributed to the distinct subcellular localization patterns of the two core proteins; the JFH1 core protein has a stronger association with LD membranes than the Jc1 core protein, which only transiently localizes near LD membranes, and thus maintaining the JFH1 protein maintains localization of PLIN2 on LD membranes and prevents its degradation.^54^ This indicates that PLIN2 is closely related to the transmission of HCV, but the effect may differ with different virus types, and is related to the degree of close contact of the HCV core protein with LDs (Fig. 4B). In liver biopsy specimens, both PLIN1 and PLIN2 were observed to localize to hepatocyte LDs and their levels correlated with specific HCV genotypes and the extent of steatosis, but not with the HCV viral load.^56^

Other studies have used siRNA to knock down *PLIN2* directly to investigate the relationship between PLIN2 and HCV infection, but found that *PLIN2* knockdown had no significant effect on HCV infection or LD morphology.^54^^,^^57^ However, when shRNA, a more potent method than siRNA, was used to reduce *PLIN2* levels in the hepatoma cell line Huh7.5, the role of PLIN2 in promoting HCV replication was uncovered, showing that the extent of HCV infection was closely linked to the degree of shRNA-induced knockdown, as PLIN2 deficiency disrupted the transport of viral core and NS5A proteins to LDs. This consequently impacted the production of infectious viral particles and decreased apolipoprotein E (ApoE) expression via lysosomal degradation, thereby reducing HCV virion assembly.^58^

## Alcohol-related liver disease

Excessive alcohol consumption is associated with the development of ALD, which can progress from hepatic steatosis to cirrhosis and HCC. Alcohol affects hepatic lipid metabolism, increasing hepatic fatty acid uptake, increasing hepatic neovascularization, attenuating mitochondrial β-oxidation, and decreasing very-low-density lipoprotein secretion, ultimately increasing hepatic lipid accumulation.^59^ Considerable effort has been made to explore the role of PLIN2 in the pathogenesis of ALD. *Plin2-*deficient mice chronically exposed to alcohol exhibited protection against hepatic steatosis, glucose intolerance, and hepatic ceramide accumulation, which may be related to the down-regulated expression of genes involved in lipogenesis and triglyceride synthesis, highlighting the critical role of PLIN2 in the development of experimental ALD.^60^

PLIN2 is known to contribute to the pathogenesis of ALD, and many studies have explored the factors that regulate PLIN2. Ceramide synthase 6 (CerS6) may act as a novel regulator of PLIN2. *In vitro* of alcoholic steatosis, CerS6 up-regulated PLIN2 expression, and inhibition of CerS6 *in vivo* reduced hepatic lipid accumulation by destabilizing *Plin2* mRNA and promoting alcoholic steatosis (the initial stage of ALD).^61^^,^^62^ Decreased expression of SET domain bifurcated 1 (*Setdb1*) in the livers of alcohol-fed mice increased *Plin2* transcription, inhibited AMPK phosphorylation, and disrupted the PLIN2-trapping CMA protein complex, ultimately stabilizing PLIN2 and impeding LD degradation.^63^ Decreased miR-150 expression acted as a compensatory response to alcohol-induced hepatic steatosis, but miR-150 overexpression exacerbated liver lipid accumulation and aggravated alcohol-induced hepatic steatosis.^64^ miR-150 functions as a pro-steatosis effector that activated *PLIN2* transcription by directly binding to overlapping RNA transcripts of the *PLIN2* promoter, thus recruiting the DNA helicase DHX9 and RNA polymerase II.^64^ These studies revealed that *CerS6*, *Setdb1*, and miR-150 could regulate PLIN2 levels in LDs during alcohol-induced steatosis, influencing the severity of alcohol-induced steatosis by affecting *PLIN2* transcription (Fig. 4C).

## Metabolic associated fatty liver disease

MAFLD, formerly known as non-alcoholic fatty liver disease (NAFLD), is a chronic, heterogeneous liver disease characterized by the accumulation of LDs in hepatocytes that can progress to liver fibrosis and HCC.^65^^,^^66^ The global prevalence of MAFLD is approximately 32.4%.^67^ In hepatocyte-specific *Plin2-*deletion mice fed a western-style obesity diet, PLIN2, which acts as a prominent scaffold protein in lipid storage structures, was crucial for the progression of MAFLD to metabolic dysfunction-associated steatohepatitis (MASH) and liver fibrosis, and *Plin2-*deletion in mice protected against diet-induced MAFLD.^68^ Another animal model induced by a high-fat diet also showed a relationship between the high-fat content and PLIN2. In a different high-fat diet-fed mouse model, hepatocyte LDs exhibited elevated lipid peroxidation levels alongside a significant increase in PLIN2.^69^ This suggests that obesity correlates with heightened hepatocellular lipid peroxidation, which may involve PLIN2-mediated accumulation of triacylglycerol and sterol ester in LDs during lipogenesis.^69^ PLIN2 could also potentially mitigate oxidative damage by sequestering harmful lipids, though further mechanistic studies are needed to confirm this role (Fig. 4D).

In addition to animal experiments, a population-specific cross-sectional study involving 142 patients with type 2 diabetes mellitus categorized as MAFLD and non-MAFLD groups found that MAFLD patients had higher circulating perilipin 2 (cPLIN2) levels than non-MAFLD patients. Subsequent analyses revealed a significant correlation between cPLIN2 and MAFLD, indicating that serum cPLIN2 may serve as an independent risk factor associated with MAFLD pathogenesis.^70^ Recent studies suggested that PLIN2 may promote the pathogenesis and pathological progression of high-fat diet-induced MAFLD.

*Plin2* deletion protected mice from diet-induced MAFLD. To elucidate the underlying mechanisms responsible for this protective effect, a western-style obesity diet-fed *Plin2*-deletion mouse model was established, and its primary hepatocytes were isolated.^57^ The study revealed that *Plin2* deletion enhanced autophagy, which increased fatty acid oxidation in hepatocytes, but was attenuated by inhibiting autophagy or ATGL.^71^ This study demonstrated that the combined action of ATGL and lipophagy drove the protective effect of hepatic *Plin2* deletion on MAFLD in mice.

## Hepatocellular carcinoma

There were approximately 747,000 HCC cases worldwide in 2019, an increase of 70% compared with 1990. China has the largest HCC burden in the world, with more than 290,000 individuals diagnosed with HCC in China in 2019.^72^ In a study comparing three types of HCC lesions, macrovesicular steatosis-HCC, microvesicular steatosis-HCC, and conventional HCC, macrovesicular steatosis-HCC had higher steatosis levels in the background liver than conventional HCC, and both macrovesicular steatosis-HCC and microvesicular steatosis-HCC had higher PLIN2 expression in LDs than conventional HCC, indicating distinct characteristics of LDs in HCC lesions compared with those in the background liver, suggesting a link between HCC steatosis and PLIN2.^73^ This indicated a potential role of PLIN2 in the pathological changes of HCC steatosis.

The effect of PLIN2 on the proliferation of HCC cells has also been studied; however, the results differed. In HCC cells and tissues, PLIN2 is frequently overexpressed and promotes proliferation by stabilizing hypoxia-inducible factor 1-α (HIF1α) protein through inhibition of the AMPK/Unc-51-like autophagy-activating kinase 1 (ULK1)-mediated autolysosome pathway.^74^ Conversely, PLIN2 has also been linked to tumor cell death under specific conditions. The increased expression of thyroid hormone receptor-interacting protein 13 (TRIP13) was discovered in both human and mouse HCC.^75^ When TRIP13 levels were reduced *in vitro*, PLIN2-coated LDs accumulated and functioned as acentrosomal microtubule-organizing centers (aMTOCs), disrupting mitotic spindle polarity and triggering tumor cell death.^75^ This process was PLIN2-dependent, though the precise mechanisms remain unclear.^75^ The contrasting findings suggest that PLIN2 plays a context-dependent dual role in HCC development. Under hypoxic conditions, PLIN2 stabilizes HIF1α to promote tumor proliferation, while in situations of TRIP13 deficiency, PLIN2-mediated LD aggregation and conversion into aMTOCs induce mitotic dysfunction and cell death (Fig. 4E). Further studies are needed to fully elucidate the regulatory mechanisms governing PLIN2's context-dependent effects in HCC.

## Potential clinical implications of PLIN2 in liver disease

**Figure 5:**
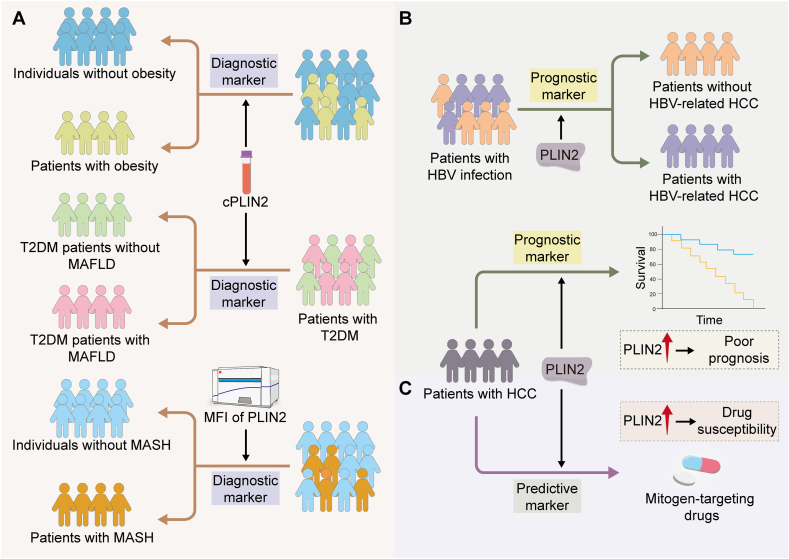
Potential applications of PLIN2 as biomarkers in liver disease. PLIN2 can serve as a diagnostic **(A)**, prognostic **(B)**, and predictive **(C)** marker in different liver diseases. cPLIN2, circulating perilipin 2; HCC, hepatocellular carcinoma; HBV, hepatitis B virus; MFI, mean fluorescence intensity; MAFLD, metabolic associated fatty liver disease; MASH, metabolic dysfunction-associated steatohepatitis; T2DM, type 2 diabetes mellitus.

PLIN2 has emerged as a versatile biomarker for liver disease and serves as a diagnostic, prognostic, and predictive marker. PLIN2 has great potential in liver disease diagnosis and prognosis prediction. In a cross-sectional study, serum cPLIN2 levels were higher in type 2 diabetes mellitus patients with MAFLD than in those without MAFLD, indicating a significant correlation between PLIN2 and MAFLD, thus suggesting its potential as an indicator of MAFLD in patients with type 2 diabetes mellitus (Fig. 5A).^70^ In addition, an analysis of cPLIN2 levels in two cohorts of elderly and young individuals revealed that cPLIN2 was strongly associated with body mass index, and after adjusting for body mass index, leptin was identified as the factor most strongly associated with the cPLIN2 levels, suggesting that leptin may be a link between fat mass and cPLIN2 (Fig. 5A).^76^ This indicates that serum cPLIN2 and leptin levels may be considered indicators of body obesity. In a multicenter cohort, the high diagnostic accuracy of the mean fluorescence intensity of PLIN2 combined with other factors for MASH suggested that using peripheral blood monocyte PLIN2 as a biomarker in liquid biopsy testing may replace invasive liver biopsy for MASH diagnosis and treatment (Fig. 5A).^77^ The specific aggregation of cofilin 1 (CFL1) and PLIN2 in tissues from HBV-related HCC patients and their positive correlation with disease severity suggested the potential of CFL1 and PLIN2 as biomarkers for both the diagnosis and prognosis of HBV-related HCC (Fig. 5B).^78^ Similarly, PLIN2 levels are frequently up-regulated in HCC cells and tissues, and increased expression has been associated with a poor prognosis, indicating that PLIN2 may be a prognostic biomarker for HCC (Fig. 5B).^74^ Moreover, beyond its prognostic role, PLIN2 may also serve as a marker indicating susceptibility to cancer therapies targeting the spindle assembly checkpoint and microtubule-toxic drugs. PLIN2 levels may help stratify tumors based on their response to specific mitogen-targeting agents for tailored treatment plans with agents such as paclitaxel and TRIP13 inhibitors, potentially enhancing treatment efficacy by predicting drug susceptibility (Fig. 5C).^75^

## PLIN2 as a potential therapeutic target in liver disease

**Figure 6:**
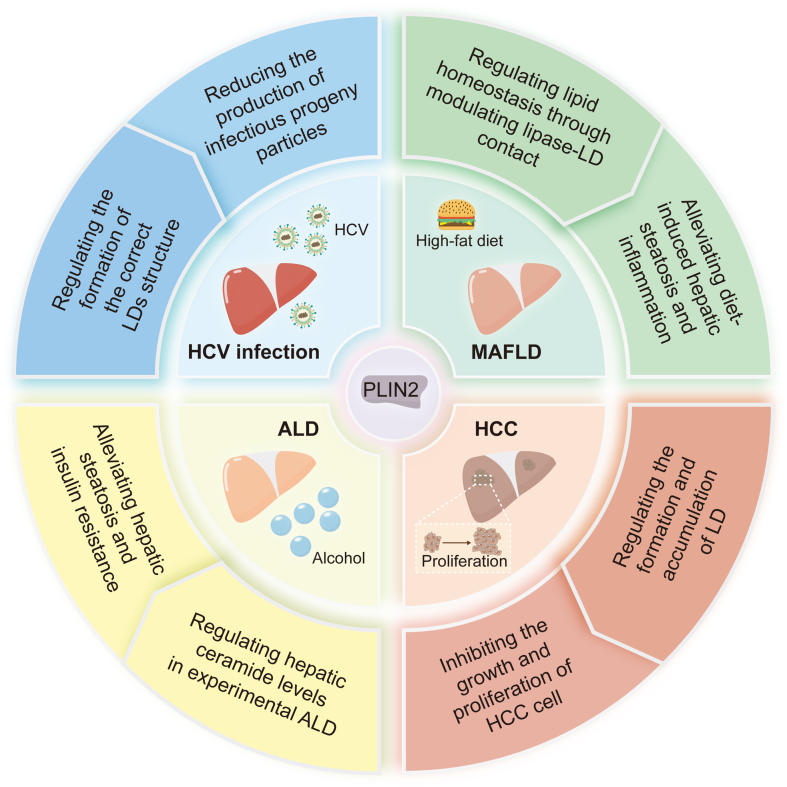
The effects of PLIN2 as a therapeutic target in liver disease. It illustrates the potential effects of PLIN2 as a therapeutic target in different liver diseases. ALD, alcohol-related liver disease; HCC, hepatocellular carcinoma; HCV, hepatitis C virus; LDs, lipid droplets; MAFLD, metabolic associated fatty liver disease; PLIN2, perilipin 2.

Although no drugs currently target PLIN2 or regulate LD activity to treat HCV infection, PLIN2 is necessary to form the correct LD structure, which is required to transport the viral core protein and NS5A to the LD surface and form functional low-density HCV particles before ApoE is incorporated.^58^ Hypothetically, pharmacological inhibition of PLIN2 could disrupt this process, potentially reducing infectious particle production (Fig. 6). As this approach would not directly act on HCV itself, it might offer theoretical advantages against resistance development. However, this remains speculative.

MAFLD/MASH is a leading cause of liver disease worldwide. MASH is an advanced form of MAFLD that is more likely to develop into cirrhosis and HCC. Lifestyle intervention remains the most important and effective strategy for preventing and controlling MAFLD progression, yet drugs specifically designed to treat MAFLD/MASH are still limited. Metabolic disorders, such as steatosis, are considered to be important in the pathogenesis of MAFLD/MASH; therefore, targeting abnormal lipid metabolism to prevent liver fat accumulation and the generation of a profibrotic environment may be an effective therapeutic strategy.^79^ Certain drugs targeting PLIN2, such as exogenous H_2_S treatment, have been shown to alleviate MAFLD by regulating lipid droplet metabolism.^80^ As an important lipid droplet-binding protein in the liver, PLIN2 stabilizes lipid storage by limiting binding between lipase and LDs (Fig. 6).^81^ *Plin2* knockout alleviated diet-induced hepatic steatosis and inflammation via phosphoethanolamine N-methyltransferase (PEMT), leading to compensatory changes in protein levels associated with phospholipid remodeling, inflammation, and endoplasmic reticulum stress, highlighting the potential of targeting PLIN2 as a therapeutic strategy in MASH.^82^ Knocking down endogenous cannabinoid receptor 1 (CB1) *in vivo* and pharmacologically antagonizing CB1 in cell culture reduced PLIN2 expression by down-regulating PPARγ expression, which was associated with lipogenesis inhibition, triglyceride synthesis, and autophagy enhancement.^83^ Based on the results of a liver tissue transcriptome data analysis, the expression of four key genes, insulin receptor substrate 2 (*Irs2*), patatin-like phospholipase domain containing 2 (*Pnpla2*), *Plin2*, and sterol regulatory element binding transcription factor 2 (*Srebf2*), was significantly up-regulated in the liver of mice with high-fat diet-induced lipid accumulation, and the expression of these genes may be key therapeutic targets and early diagnostic markers of MAFLD.^84^ In mice lacking endogenous PLIN2, the expression of PLIN2-Ser251, compared with that of human wild-type PLIN2-Ser251, alleviated hepatic steatosis induced by a high-fat, high-fructose, high-cholesterol diet, with lower expression of polyunsaturated fatty acid triglycerides and cyclooxygenase genes and reduced liver oxidative stress.^85^ PLIN2-Ser251 may be a novel LD protein target for treating hepatic steatosis.

In addition to MAFLD/MASH, related studies have demonstrated that PLIN2 is a potential target of certain drugs, such as dietary quercetin, for preventing steatosis in ALD by regulating LD metabolism.^86^ The increase in hepatic ceramide levels in experimental ALD was prevented when PLIN2 was deficient.^60^ In contrast, the onset of hepatic steatosis and insulin resistance in experimental ALD was associated with an increase in long-chain hepatic ceramide levels and an up-regulation of PLIN2 expression,^87^ suggesting that PLIN2 may mediate cellular ceramide metabolism and insulin resistance in ALD, thus rendering PLIN2 a potential target for the treatment and prevention of ALD (Fig. 6).

Reprogrammed lipid metabolism has been recognized as a hallmark of cancer.^35^ Metabolic adaptation occurs in cancer cells (including HCC) in response to increased metabolic demands. Lipid biosynthesis is a specific adaptation pathway. Studies have shown that lipid biosynthesis and desaturation are critical for HCC survival^88^; however, there is a lack of cancer treatment strategies that regulate lipid metabolism. Moreover, enhanced lipogenesis, increased lipid content (either free or stored in LDs), and lipid-dependent catabolism promote therapeutic desensitization and drug-resistant phenotypes of tumor cells treated with chemotherapy or targeted therapy. This suggests that lipid metabolism reprogramming may potentially delay or prevent the development of resistance to anti-cancer therapies.^89^ Stored lipids in LDs can serve as an energy source that promotes tumor invasion and migration through β-oxidation.^90^^,^^91^ Moreover, promoting LD degradation in lysosomes can inhibit HCC cell growth.^92^ The expression of PLIN2 is frequently up-regulated in HCC and promotes cell proliferation.^74^^,^^75^ Targeting PLIN2 may improve existing therapies or create new opportunities for HCC treatment (Fig. 6).

## Conclusions and future perspectives

PLIN2 is a typical LD-related protein that regulates fat metabolism, lipid droplets, and the onset and progression of liver disease. PLIN2 regulates the contact between LDs and other organelles, preventing the further transport of lipids, thereby stabilizing lipids in LDs and promoting the formation and maturation of LDs. In viral hepatitis, the expression of PLIN2 is significantly increased, which is related to the production of HCV transmissible particles, and can affect liver lipid metabolism after HBV infection. Regardless of the cause of fatty liver disease, diet or alcohol, increased PLIN2 expression is closely related to the degree of fat and liver damage. Additionally, PLIN2 expression in patients with HCC was significantly increased in tumors, and pathological changes in the liver were closely related to tumor cell proliferation.

PLIN2 may be a potential biomarker, with promising clinical applications in diagnosis, drug susceptibility, and prognostic assessment. Determining the level of PLIN2 can assist in the early diagnosis and monitoring of liver disease, including in obesity. PLIN2 may also be an important indicator for evaluating the sensitivity of patients with HCC to therapeutic drugs and predicting their prognosis. In addition, PLIN2 may be a potential target for treating liver disease. Modulating PLIN2 expression and functions may regulate LD metabolism and fat oxidation, thereby slowing or even stopping the pathological process of fatty liver formation. Additionally, regulating PLIN2 expression may affect the growth and spread of HCC cells, providing new approaches for treating HCC.

In summary, PLIN2 plays a key role in the pathogenesis and progression of liver disease and has broad clinical prospects. Although the importance of PLIN2 in liver disease has been widely recognized, its exact mechanism of action is not fully understood. By studying the specific mode of action of PLIN2 in fat metabolism, hepatitis development, and tumor cell proliferation, its function in liver disease can be further elucidated, and new approaches and strategies for diagnosing and treating liver disease can be developed.

## CRediT authorship contribution statement

**Xuwen Xiang:** Writing – review & editing, Writing – original draft, Visualization, Software, Investigation. **Jing Chen:** Writing – review & editing, Writing – original draft, Project administration, Investigation, Funding acquisition, Conceptualization. **Lin Che:** Writing – review & editing, Writing – original draft, Visualization, Investigation, Funding acquisition, Conceptualization.

## Funding

This work was supported by the 10.13039/501100001809National Natural Science Foundation of China (No. 82304180, 82103859), Fujian Province Natural Science Foundation (China) (No. 2023J011602), 10.13039/501100021171Guangdong Basic and Applied Basic Research Foundation (No. 2024A1515010893), Project funded by 10.13039/501100002858China Postdoctoral Science Foundation (No. 2022M713585, 2024T171083), Fujian Provincial Health and Medical Innovation Research Project (2024CXB022), and Xiamen Municipal Science and Technology Program for High-Quality Development in Health and Medicine–Medical Innovation Project (2024GZL-CX23).

## Conflict of interests

The authors declared no conflict of interests.
